# Superoxide Dismutase Administration: A Review of Proposed Human Uses

**DOI:** 10.3390/molecules26071844

**Published:** 2021-03-25

**Authors:** Arianna Carolina Rosa, Daniele Corsi, Niccolò Cavi, Natascia Bruni, Franco Dosio

**Affiliations:** 1 Department of Scienza e Tecnologia del Farmaco, University of Turin, Via P. Giuria 9, 10125 Turin, Italy; daniele.corsi94@outlook.com (D.C.); niccolo.cavi@edu.unito.it (N.C.); franco.dosio@unito.it (F.D.); 2 Istituto Farmaceutico Candioli, Strada Comunale di None, 1, 10092 Beinasco, Italy; natascia.bruni@candioli.it

**Keywords:** antioxidant, superoxide dismutase, supplementation, detoxification

## Abstract

Superoxide dismutases (SODs) are metalloenzymes that play a major role in antioxidant defense against oxidative stress in the body. SOD supplementation may therefore trigger the endogenous antioxidant machinery for the neutralization of free-radical excess and be used in a variety of pathological settings. This paper aimed to provide an extensive review of the possible uses of SODs in a range of pathological settings, as well as describe the current pitfalls and the delivery strategies that are in development to solve bioavailability issues. We carried out a PubMed query, using the keywords “SOD”, “SOD mimetics”, “SOD supplementation”, which included papers published in the English language, between 2012 and 2020, on the potential therapeutic applications of SODs, including detoxification strategies. As highlighted in this paper, it can be argued that the generic antioxidant effects of SODs are beneficial under all tested conditions, from ocular and cardiovascular diseases to neurodegenerative disorders and metabolic diseases, including diabetes and its complications and obesity. However, it must be underlined that clinical evidence for its efficacy is limited and consequently, this efficacy is currently far from being demonstrated.

## 1. Introduction

**Figure 1:**
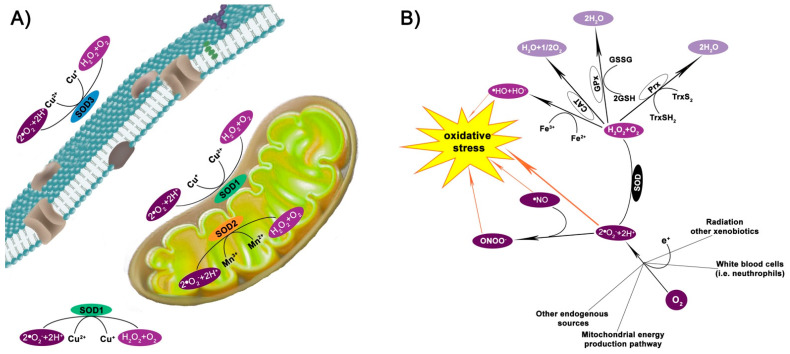
Superoxide dismutase enzymes. (**A**) Superoxide dismutases (SODs) are metalloenzymes constitutively expressed in eukaryotes: SOD1 is a Cu, Zn-SOD and is present in the cytosol and the mitochondrial intermembrane; SOD2 is a Mn-SOD localized in the matrix and inner membrane of mitochondria; SOD3 is a Cu, Zn-SOD expressed in the extracellular compartment. Nevertheless, all three forms catalyze the conversion of the superoxide anion free radical (^•^O_2_^−^) into hydrogen peroxide (H_2_O_2_). (**B**) In detail, SOD converts the ^•^O_2_^−^, generated in several cellular insults/metabolism, into H_2_O_2_ and molecular oxygen (O_2_). The resulting H_2_O_2_ may undergo reduction to water via catalase (CAT), glutathione peroxidases (GPx), or thioredoxin (Trx)-dependent peroxiredoxin (Prx). Otherwise, H_2_O_2_ originates ^•^OH via the Fenton reaction in the presence of Fe^2+^. ^•^O_2_^−^ may also react with ^•^NO originating the oxidant and nitrating agent peroxynitrite (ONOO^−^), which further contributes to oxidative-stress damage. GSH = glutathione; GSSG = glutathione disulfide; TrxSH_2_ = reduced thioredoxin; TrxS_2_ = oxidized thioredoxin.

Superoxide dismutases (SODs) are metalloenzymes found in eukaryotes and some prokaryotes and as shown in Figure 1A, they are localized in the cytosol and the mitochondrial intermembrane (Cu, Zn-SOD or SOD1), the mitochondrial matrix and inner membrane (Mn-SOD or SOD2) [1], and extracellular compartment (Cu, Zn-SOD or SOD3) [2].

Since their discovery by Joe McCord and Irwin Fridovich [3], their role as a major antioxidant defensehas been firmly recognized [4]. The work by I. Fridovich and collaborators was crucial in defining the role of oxidant/antioxidant processes in ischemia/reperfusion-associated pathologies in humans and animal models [5,6].

SOD catalyzes the conversion of the superoxide anion free radical (^•^O_2_^−^) to hydrogen peroxide (H_2_O_2_) and molecular oxygen O_2_ (Figure 1A,B). Subsequently, H_2_O_2_ is reduced to water by the catalase (CAT) enzyme, glutathione peroxidase (GPx), and/or thioredoxin (Trx)-dependent peroxiredoxin (Prx) enzymes (Figure 1B). H_2_O_2_ may also generate another reactive oxygen species (ROS), the hydroxide ion (^•^HO) via the Fenton reaction in the presence of Fe^2+^ (Figure 1B).

H_2_O_2_ is an essential sensor in redox metabolism. Its levels are critical to oxidative stress: under physiological conditions, when H_2_O_2_ intracellular concentration are 1–10 nM, it mediates the stress response involved in the physiological and adaptive processes called oxidative eustress; higher concentrations (more than 100 nM) are responsible for the so-called oxidative distress, in which the evoked inflammatory response leads to cell damage [7,8]. Considering the endogenous antioxidant system involved in H_2_O_2_ production and removal, a parallel dual role, physiological and pathological, can also be recognized for all the enzymes involved. SOD activity may therefore have a double and opposite meaning [9]: firstly, it is an antioxidant enzyme when its activity is coordinated with either the CAT, GPx or Prx/Trx enzymes, which avoid H_2_O_2_ accumulation by neutralizing it into H_2_O; secondly, SOD may act as a pro-oxidant as H_2_O_2_ can overaccumulate, leading to ROS overproduction and cell toxicity [7].

Accordingly, a bell-shaped dose-response curve describes the protective effects of SOD on isolated heart preparation, with low doses (up to 5 μg/mL in the perfusate) protecting, and high doses (50 μg/mL in the perfusate) exacerbating reoxygenation-induced injury [10]. However, when SOD activity increases, the enhanced levels of H_2_O_2_ trigger the upregulation of CAT [11] and/or GPx [12], with a final antioxidant balance as a compensatory and defense response strategy.

SODs are also involved, at least partially, in detoxification from the oxidant and nitrating agent peroxynitrite (ONOO^−^), which is formed from the reaction between ^•^NO and ^•^O_2_^−^ (Figure 1B). ONOO^-^ rapidly forms reactive free radicals upon reaction with CO_2_ [11]. SOD also prevents this detrimental event.

On this basis, it is universally recognized that SOD is the first line of defense against the toxicity of ^•^O_2_^−^ because catalyzing the dismutation of two molecules of ^•^O_2_^−^ to hydrogen H_2_O_2_ and O_2_ limits the ^•^O_2_^−^ availability. Low and diminished SOD activity has been associated with a significant risk of oxidative stress, resulting in disease, such as hypertension, hypercholesterolemia, atherosclerosis, diabetes, heart failure, stroke and other cardiovascular diseases [12,13]. Therefore, it has been suggested that the antioxidant properties of SOD supplementation are useful in a variety of pathophysiological conditions, from protecting the immune system to the prevention of aging [14]. The consumption of natural sources of SOD, such as cabbage, Brussels sprouts, wheatgrass, barley grass and broccoli has been encouraged [15].

The use of SOD as a drug may be advantageous in terms of the quantity and duration of the pharmacological effect, compared to other antioxidants. Indeed, SOD supplementation may trigger the endogenous antioxidant machinery to neutralize a free radical excess without being consumed upon ROS detoxification. By contrast, non-enzymatic antioxidants, such as glutathione (GSH), are known to be depleted [16]. However, pharmacological treatment using exogenous SOD administration is not yet an established clinical practice, and usually dietary supplementation is pursued. Indeed, efficacy depends on the source of SOD. Although there is a lack of head-to-head studies, a study in rats has demonstrated that human and bovine SODconferred higher pharmacological activity that the rat enzyme [17].

Moreover, the treatment of human diseases with the human enzyme may not yield beneficial effects. Bovine SOD, known as orgotein, was usually preferred. However, it can be limited by its intramuscular administration, administration frequency (2~3 times weekly) [9], and possible toxicity, caused by the presence of 20% impurities (albumin and chymotrypsin are the primary contaminants), in the pharmaceutical preparation that may result in immediate hypersensitivity reactions [18], and other side effects, including allergy [16]. Orgotein, marketed for the treatment of a range of inflammatory diseases, was withdrawn from European countries [18], due to allergic reactions, and limited to veterinary use in the US.

Over time, plant-extracted SOD became the alternative. Cantaloupe-melon-(*Cucumis melo* L.C.)-derived SOD, SODB, which offers the advantage of a high SOD concentration (100 U/mg) and low contents of other antioxidants, such as CAT (10 U/mg) and GSH (1 U/mg), is one of the most commonly used [19,20]. However, the oral bioavailability of this form of SOD is still very low, according to the general pharmacokinetics principle of drugs, and this is because of its high molecular weight, which affects cellular uptake [21], and the low pH and high proteolytic activity in the digestive tract [22]. As natural SOD is an exogenous protein, we can hypothesize that it may induce antibody formation (anti-drug antibodies ADA). However, considerable experience with the infusion of proteins as drugs for therapeutic purposes has indicated that there is only a marginal reduction in their effect and no clinically demonstrated toxicity.

Thus, the use of SOD mimetics and new delivery systems to protect SOD are under investigation [23]. SOD mimetics are intended to overcome the limits of natural SOD enzymes. They have better pharmacokinetic properties and some pharmacodynamic differences, with negligible antigenicity potential. Indeed, SOD mimetics have a low molecular weight, more stability and a long-circulating half-life, guaranteeing a better pharmacokinetic profile. Moreover, they have a different dose–response curve; natural SOD displays a bell-shaped dose-dependent curve, while most SOD mimetics have a dose-proportional response [24]. Finally, their mechanism of action is far beyond that of ^•^O_2_^−^ scavenger activity alone, as discussed below.

This paper aimed to provide an extensive review of the possible uses of SOD in different human diseases and explore the current pitfalls in development processes to solve the bioavailability issues. Selection was based on orgotein indications and included neurological, cardiovascular, respiratory, gastrointestinal, renal, skin, metabolic and ocular diseases. We are aware that cancer is a meaningful field of application for SOD. However, we stress that oncology is far beyond our expertise and has been extensively reviewed in I. Batinic-Haberle and coll. (2018) [25], I. Batinic-Haberle and I. Spasojevic [26], and I. Batinic-Haberle and M. E. Tome [27]. We therefore carried out a PubMed query starting with the keywords “SOD”, “SOD mimetics”, and “SOD supplementation” that included papers published in the English language, between 2012 and 2020, on the potential therapeutic applications of SOD, including detoxification strategies.

## 2. Mechanism of SOD Induction and Inactivation

The three isoforms of SOD show differences in their protein structures, metal cofactor requirements, subcellular localization (Figure 1), and tissue distribution. Human SOD1 is an homodimer of 88 kDa that is encoded by a gene on chromosome 21q22 [28]. SOD2 is a smaller homotetramer protein of 32 kDa, encoded by a gene on chromosome 6q25.3 [29]. Finally, SOD3 is an homotetramer glycoprotein of 135 kDa encoded by a gene on chromosome 4 [30].

Some unique transcription factors that play specific regulatory roles have been described [31]. However, all three SOD isoforms share the presence of binding sites for several transcription factors, such as the Nuclear Factor (NF)-κB, the specificity protein (Sp)-1, CCAAT-Enhancer-Binding Proteins (C/EBP), and the activator proteins (AP)-1 and-2, which exert similar effects on the regulation of all three *SOD* genes [31,32,33]. A prominent role has been recognized for nuclear factor erythroid 2-related factor 2 (Nrf2). The first evidence of the relationship between SOD1 and Nrf2 dates back to 2005, when the presence of the SODG93A mutation was associated with a reduction in Nrf2 mRNA [34]. Nrf2 translocates to the nucleus from the cytoplasm following binding with the Kelch-like ECH-associated protein 1 (Keap1). Keap1 is a cysteine-rich protein that interacts with ROS and promotes both the nuclear translocation and the ubiquitination and degradation of Nrf2. In the nucleus, Nrf2 forms a complex with Maf (musculoaponeurotic fibrosarcoma) proteins. It binds the antioxidant responsive elements (AREs) [35] at the sequence located in the promoter region between −356 and −330 from the transcription start site of *sod1* [36]. 

The Keap1/Nrf2 pathway regulates the expression of many antioxidant genes besides SODs, such as those encoding for CAT, GPx, NAD(P)H-quinone oxidoreductase 1, GSH-S-transferase, Prx, ferritin and heme oxygenase-1 (HO-1) [37]. Interestingly, the Keap1/Nrf2 pathway can be considered the effector of the SOD mimetic mechanism of action. Indeed, SOD mimeticsalter the cysteine oxidation/protein S-glutathionylation cycle. These compounds cause the oxidation of the thiols of the peptide cysteine of Keap1, thus inducing Nrf2 activation and leading to SOD overexpression [27].

The Keap1/Nrf2/HO-1 axis and its link to SOD expression have been well characterized, and are based on the complementary function of SOD and HO-1; the first produces H_2_O_2_ and the second catalyzes the rate-limiting step in the breakdown of heme to bilirubin [38], which is known to remove ROS, including ^•^OH, singlet oxygen and ^•^O_2_ [39]. Accordingly, the subsequent induction of SOD2 and HO-1 has been identified as the mechanism by which the Nrf2-ARE inducer tert-butylhydroquinone protects mitochondria that are exposed to oxidative stress [40], and astrocytes that are damaged by lanthanum chloride [41]. Moreover, Nrf2/HO-1 has been demonstrated to confer protection from doxorubicin-induced mitochondrial damage by upregulating antioxidant genes, including SOD2 [42]. Similarly, cobalt protoporphyrin, a potent inducer of the HO-1 protein and activity, increased SOD3 expression in rat aorta, possibly via the activation of the mitogen-activated protein kinase (MAPK) pathway [43]. Nrf2 is a direct downstream target of MAPK, like ERK [44]. Accordingly, the Nrf2/ERK signaling pathway has been implicated in the upregulation of the gene expression of HO-1 and SOD1 by fucoidan, a sulfated polysaccharide found in edible brown algae [45]. However, in a study by M. Dell’Orco and coll. (2016), Nrf2 does not appear to be associated with SOD1 in human neuroblastoma SH-SY5Y cells that are exposed to H_2_O_2_ [46]. Considering the role of Keap1/Nrf2 in SOD expression, the Nrf2 activators, or Keap1 inhibitors [47], should be included between the SOD inducers. Among them, the peroxisome proliferator-activated receptor (PPAR)γ is particularly attractive. Indeed, it could regulate SOD expression both directly through its association with the PPAR responsive element of the SOD promoter region, and indirectly inducing the expression of Nrf2, HO-1, CAT, and GPx-3 [48]. In particular, between Nrf2 and PPARγ, a positive feedback loop reinforcing the antioxidant response is established: Nrf2 through the ARE region present on the PPARγ promoter may directly upregulate PPARγ expression and PPARγ may in turn regulate the Nrf2 interacting with a PPAR responsive element [49].

Another interesting axis in SOD transcriptional regulation can be found in the phosphoinositide 3-kinase (PI3K)/AKT/NF-κB/transcription factors of the forkhead box, class O (FOXO) axis, which has been reported to exert antioxidant effects by increasing SOD expression. Indeed, the PI3K/Akt pathway induces SOD1, SOD2 and SOD3 expression [50,51,52], as well as HO-1 [53,54]. The activation of the PI3K/AKT axis inversely regulates the distribution of NF-κB and FOXO transcription factors; FOXO factors are phosphorylated and displaced from the nucleus to the cytoplasm, while NF-κB translocates to the nucleus, activating antioxidant genes, including *SOD*s [50]. Again, PPARγ can participate: it may increase FOXO activity through the activation of AKT and NF-κB transrepression [55]. Interestingly, the role of the NF-κB-SOD axis in homeostasis through the NF-κB p65 subunit translocation is well documented and has been implicated, for instance, in the endotoxin-induced stress [56]. However, a vicious loop can be identified between SOD and NF-κB: the IKKβ/NF-κB signaling pathway regulates SOD2 expression through p53, and p53 transcription is in turn dysregulated by SOD2, causing the upregulation of IKKβ. This loop may be detrimental to the progression of tumorigenesis. Indeed, SOD2 expression was positively associated with pathologic tumor stages and negatively correlated with overall survival in nasopharyngeal carcinoma [57] or lung adenocarcinoma [58].

In addition to transcriptional regulation, epigenetic and post-transcriptional regulation can also contribute. Epigenetic regulation is primarily associated with SOD expression and activity in cancer. The most documented epigenetic regulation involves the promoter methylation of the SOD2 gene [59]. It has recently been demonstrated that the deacetylation of histones at its promoter reduces *sod3* expression in old lung fibroblasts. Accordingly, histone deacetylase inhibitors were able to preserve *sod3* expression [60]. On the other hand, in THP-1, histone H3 and H4 acetylation regulates *sod3* expression during differentiation, while DNA methylation is responsible for sod3 silencing in human peripheral blood mononuclear cells (PBMCs) [61]. Post-transcriptional regulation is responsible for the rapid modulation of SOD expression and includes: (i) phosphorylation; (ii) amino acid modification, such as lysine acylation (including sumoylation, ubiquitination and glycation); (iii) redox modifications, such as oxidation, glutathionylation and cysteinylation; (iv) s-acylation; and (v) nitration [62,63,64].

Apart from expression regulation, SOD activity also depends on the presence of the associated metals. These mechanisms have been extensively reviewed by Culotta et al. (2007), Fukai and Fukai (2011), and Hatori and Lutsenko (2016). Briefly, while SOD1 and SOD3 exist as apoenzymes that are activated post-transcriptionally by copper insertion (without new protein synthesis), metal insertion for SOD2 cannot occur post-translationally. Indeed, manganese insertion only occurs in newly synthesized SOD2, when the pre-sequence for mitochondrial targeting at the *N*-terminus is still present. Subsequently, manganese trafficking to SOD2 is driven by the Smf2p manganese transporter and Mtm1p, which are members of the mitochondrial carrier family of transporters. SOD2 is therefore imported into mitochondria and cleaved into the mature form. Conversely, SOD1 activation occurs post-transcriptionally via a 4-step process that involves the copper chaperone for SOD1 (CCS). CCS docks with and transfers copper to the disulfide-reduced SOD1. The disulfide is essential for both structural stabilization and functional activation, allowing the dimeric state to form [65,66]. Finally, SOD3 is loaded with copper via a copper chaperone antioxidant-1 (Atox1) pathway [67,68,69]. However, Atox1 is not sufficient, and the Menkes ATPase, ATP7A, is required to deliver the copper to SOD3 at the trans-Golgi network [66]. The activation of SOD leads to the conversion of ^•^O_2_^-^ to H_2_O_2_ and O_2_, as described in the above section and depicted in Figure 1. However, SOD1 can also act as a transcription factor. Indeed, H_2_O_2_ induces SOD1 translocation to the nucleus following association with the Mec1/ATM effector Dun1/Cds1 kinase and phosphorylation. Once in the nucleus, SOD1 regulates the expression of various oxidative stress-responsive genes that are known to confer resistance to oxidative stress, DNA damage repair and replication stress relief [70]. Moreover, upon binding to DNA, SOD1 regulates the ROS-responsive expression of functional genes, including oncogenes and amyotrophic lateral sclerosis-linked genes [71]. Finally, SOD1 has also been reported to activate the muscarinic M1 receptor, thus inducing AKT and ERK phosphorylation in neuroblastoma SK-N-BE cells [72].

As SOD activity depends on the associated metals, it is reasonable to assume that any perturbation of the enzyme structure that causes their release is responsible for the inactivation of the enzyme. Accordingly, using a zebrafish model, it has been demonstrated that lead forms a complex with SOD1 via an electrostatic effect. Consequently, the metal enters the active channel of SOD, hindering substrate access. Therefore, copper and zinc are released from the SOD1 active site [73]. Moreover, it is well known that the reaction of peroxynitrite with the metal center of the enzyme is responsible for SOD inactivation. In particular, both SOD1 and SOD2 react directly with peroxynitrite; SOD1 is subjected to histidinyl radical formation [74], and SOD2 is subjected to tyrosine nitration [75].

## 3. The Role of SOD: What We Have Learned from Knock-Out (KO) Mice

SOD’s role in oxidative stress defense means that its role in other pathophysiological contexts is inferable. Accordingly, the use of SOD supplements or SOD mimetics in several potential therapeutic applications is currently under investigation. Each of these possible therapeutic indications for SOD is mainly based on the use of transgenic mice. Indeed, mice that lack either SOD1, SOD2 or SOD3 have helped us to understand the relative role of each isoform in fertility, mortality/survival and the development of specific diseases. The very first difference between SOD1, SOD2 and SOD3 is in terms of survival. Homozygous mice that lack SOD2 (SOD2^−/−^), and not SOD1 or SOD3, show a dramatic phenotype that affects lifespan, with death occurring: (i) within the first 10 days with dilated cardiomyopathy, the accumulation of lipids in the liver and skeletal muscle, and metabolic acidosis [76]; or (ii) within the first 3 weeks with severe anemia, the degeneration of neurons in the basal ganglia and brainstem, and progressive motor disturbances, characterized by weakness, rapid fatigue and circling behavior [77]. Accordingly, the homozygous missense variant, c.542G > T, p.(Gly181Val), in SOD2 may lead to toxic increases in the levels of damaging oxygen radicals in the neonatal heart, which can result in rapidly developing heart failure and death [78]. As SOD2^−/−^ die in 2~3 weeks [76,77], heterozygous SOD2 (SOD2^+/−^) mice and alternatively, conditional KO mice, in which deletion involves individual tissues, have been generated [79]. Thanks to these experimental models, it is clear that the contribution of SOD to homeostasis is tissue-specific: heart/muscle-specific SOD2 KO shows a reduced lifespan, with several electrophysiological abnormalities occurring [80]; T cell-specific SOD2 KO demonstrates a compensatory phenotype, in which other mechanisms may compensate for any loss of function; while liver-specific SOD2 KO does not show a phenotype, with the tissue appearing unaffected by SOD2 loss [79]. Platelet content and function were not affected by SOD2^+/−^ phenotype, with no difference being observed between KO and wild-type mice in the tail-bleeding or arterial-thrombosis indices. Similar results have also been obtained when comparing these two phenotypes for outcomes in both sepsis and autoimmune inflammatory arthritis models [81].

Interestingly, postnatal motor neuron SOD2 KO shows no signs of oxidative damage up to 1 year after birth. These data suggest that postnatal motor neurons are resistant to oxidative-stress damage, although the disorganization of the distal nerve axon occurs [82]. Mammary-gland development is also not affected by SOD deletion; postnatal mammary gland SOD2 KO mice show no changes in pre- and post-pregnancy developmental structures and mammary-gland function [83]. 

In SOD2^+/−^ animals, enzymatic activity is decreased by 30–80% depending on the specific tissue [84]. This defect has been correlated with an increase in oxidative damage to mitochondria, but not to cytosolic proteins or nuclear DNA [85]. At 6 months, SOD2^+/−^ mice show behavioral impairments involving learning and memory processes, and alterations in glutamatergic synaptic transmission with a decrease in the *n*-methyl-D-aspartate (NMDA) receptor [86]. A clear phenotype has also been recognized in SOD1 KO mice. In this case, homozygous KO females have reduced fertility due to an increase in embryonic lethality, although normal ovulation and conception were observed [87]. These mice are healthy, although they have reduced survival time (mean lifespans of 20.8 ± 0.7 compared to 29.8 ± 2.1 months for the wild-type counterpart), with a higher incidence (79% of KO animals) of hepatocellular carcinoma development [88]. SOD1 has long been linked to age-associated diseases because SOD1 deletion leads to different phenotypes that mimic accelerated aging [89]. For instance, SOD1^−/−^ senescent mice show the decreased production of both stimulated and non-stimulated tears due to several alterations in the lacrimal gland, including: the atrophy of acinar units; fibrosis; infiltration of T-cells, monocytes and neutrophils; increases in apoptotic cells; and signs of epithelial-mesenchymal transition [90]. At 1 year of age, SOD^−/−^ mice develop cortical lens opacity, and within 1 more year, they showed reduced GSH content at the lens level [91]. Accordingly, a study of 415 cataract patients has demonstrated an increased risk of cataracts in patients that are polymorphic for SOD1 due to a reduced capacity to scavenge superoxide radicals in lenses [92]. Moreover, serum SOD activity has been observed to be significantly reduced in 60 patients with newly diagnosed senile non-pathologic cataracts [93]. In contrast, the SOD2^+/−^ phenotype was not related to age-related cataract development [94], suggesting that SOD1 may have a more detrimental effect on ageing. SOD1 deletion is also associated with cochlear degeneration over time; null mice developed early age-related hearing loss with spiral ganglion cell degeneration at 7–9 months of age [95]. Notably, SOD2 has also been found to be involved in hearing loss. Indeed, SOD2^+/−^ mice have shown significant outer hair cell damage in cochlear turns, and their response to post-noise exposure (120 dB at 4 Hz for 4 h) at 7 and 14 days was worse than that of their wild-type counterparts [96].

Notably, SOD1 KO mice display other features of aging apart from age-related hearing loss, and these include frailty, which is a clinical syndrome highly prevalent in old age that presents at least three of the following criteria: unintentional weight loss; exhaustion; weakness: slow walking speed; and low physical activity [97]. SOD1^−/−^ mice exhibit weight loss, weakness, low physical activity and exhaustion, while inflammation and sarcopenia develop in parallel [98]. Again, a similar effect is evoked by SOD2 deletion, with SOD2^+/-^ mice showing a reduction in work-to-exhaustion that is correlated with whole-body oxygen consumption [99]. A loss of muscle mass and function is one of the most prominent aging phenotypes shown by SOD1^−/−^ mice [100]. The importance of SOD1 in motor neuron degeneration is also confirmed by the demonstrated association between SOD1 defects in skeletal muscle and amyotrophic lateral sclerosis (ALS). SOD1 mutation, leading to reduced enzyme activity, is one of the key pathological events in ALS [101], and mice that express the SOD^G93A^ mutation are the most commonly used model for this disease [102]. Other mutations of SOD1 have also been recognized in ALS, although their significance in development and penetrance differs. For instance, the SOD1 G93D mutation caused a slowly developing lower motor neuron disease with reduced penetrance [103]. On the other hand, the mutation c.271G > A, which leads to the substitution of asparagine with aspartate at position 90, seems to be associated with the rapid progression and a prominent pain syndrome [104]. Moreover, A. Canosa and coll. (2018) have reported the presence of a heterozygous novel frameshift SOD1 mutation (p.Ser108 LeufsTer15), which was predicted to cause premature protein truncation in a sporadic ALS patient. This mutation could have two different consequences: (i) less active SOD1; and (ii) a less charged protein with a higher propensity to aggregate. In both cases, the result would be an increase in oxidative damage [105].

Finally, SOD1^−/−^ mice are more susceptible to paraquat toxicity [87], and motor neuron loss after axonal injury [106].

By contrast, mice that lack SOD3 have normal development and remain healthy until at least 14 months of age without the compensatory induction of other SOD isoenzymes [107]. However, their survival time was significantly affected by exposure to >99% oxygen as severe lung edema developed [107]. These data, combined with the results of gene-array screening in SOD3^−/−^ mice [108], suggest that compensatory mechanisms occur, including the unbalance of the expression of genes involved in cell signaling, inflammation and gene transcription (37 are upregulated and nine downregulated) [108]. Like SOD1, SOD3 has also been implicated in some age-related dysfunctions. For instance, both SOD-3 and SOD1 appear to have functions in preserving corneal endothelial integrity in aging [109]. Indeed, SOD3^−/−^ mice have shown the early (starting from month 2) spontaneous age-related loss of endothelial cells in the cornea and increased susceptibility to acute inflammatory endothelial damage [110]. By comparison, the corneal endothelial cells in SOD1/3^−/−^ mice have shown more irregular morphology at an older age, suggesting they have a more vulnerable corneal endothelium [109].

SOD3^−/−^ mice of 22 months have displayed reduced transforming growth factor beta (TGF-β) levels and, consequently, a lower differentiation of fibroblasts into myofibroblasts, which results in delayed wound closure, reduced neovascularization and increased neutrophil recruitment. These results suggest that reduced levels of cutaneous SOD3 in aged mice may contribute to the impaired wound healing response in aged skin [111]. By contrast, only a slight increase in inflammatory variables and fibrosis were found in lungs from 2-year-old SOD3^−/−^ mice, compared to their wild-type counterparts [112]. However, the response of SOD3^−/−^ mice to ovalbumin (OVA) challenge resulted in severe allergic asthma [113]. Interestingly, SOD3^−/−^ mice seem to be more prone to developing injury at the inner retina and may be more susceptible to vitreoretinal diseases, including diabetic vitreoretinopathy. Indeed, SOD3^−/−^ mice present higher oxidative stress markers at the vitreoretinal interface and signaling abnormalities within the inner retina [114]. SOD3^−/−^ mice have recently been used to study the contribution of oxidative stress to proteinuric kidney diseases. A study by R.J. Tan and coll. (2015) has demonstrated that SOD3^−/−^ mice are more susceptible to renal injury in an Adriamycin-(ADR)-induced nephropathy model [115].

## 4. SOD as a Detoxification Strategy

Oxidative stress is the most common mechanism of xenobiotic toxicity. For instance, heavy metals, such as mercury, arsenic and lead, induce oxidative stress by promoting the production of ROS and reactive nitrogen species (RNS). These metals may replace the transition metals, such as Zn and Cu, which are required for SOD catalytic function, and inhibit their function [13]. Various chemicals can affect the balance between pro-oxidant challenge and antioxidant defenses by enhancing ROS and/or RNS formation and by depressing their removal [116].

Due to its role in limiting the formation of ROS and RNS and the consequent oxidative-stress damage, the availability of SOD as an antidote for xenobiotic toxicity would be a therapeutic advantage.

As SOD2^+/−^ mice have been used as an experimental model to investigate of the role of mitochondrial toxicity in troglitazone-induced liver injury [117], SOD2 has been postulated to be a key enzyme against the hepatotoxicity of some drugs and chemicals [118]. For instance, SOD2 is inactivated by protein nitration during paracetamol hepatotoxicity [119]. Furthermore, partial SOD2 deficiency and inactivation have been associated with increased liver injury [120,121,122]. It has therefore been hypothesized that increasing SOD2 expression/activity might have a beneficial effect. This strategy has been pursued using nitroxide mito-tempo, which is a compound that combines piperidine nitroxide (tempo or tempol) with triphenylphosphonium (TPP^+^), which is a membrane-permeant cation that accumulates within mitochondria thanks to membrane potential [123], tempol [124], and the Mn pyridoxyl ethyldiamine derivative (MnPLED) mangafodipir (MnDPDP) [125]. Mito-tempo and tempol are both nitroxides and their classification as SOD mimetics is controversial [24,126].

The promising results obtained in C57 BL/6 J mice with paracetamol overload (300 mg/kg i.p.) [127], and in BALB/c mice with paracetamol (1000 mg/kg i.p. or 500 mg/kg p.o.)-induced acute liver failure [125], have led to a successful evaluation of the safety and tolerability of another MnPLED SOD-mimetic, calmangafodipir [Ca_4_Mn(DPDP)_5_], in combination with *n*-acetylcysteine (the gold standard antidote for paracetamol toxicity) for paracetamol overdose in humans [128]. Thus far, calmangafodipir has been reported among the established and emerging therapies against paracetamol hepatotoxicity in a recent review [129].

Due to its beneficial effects on hepatotoxicity, SOD2 has also been proposed as an antidote against carbon tetrachloride (CCl_4_) intoxication. The CCl_4_ metabolic process in the liver gives rise to two active microsomal radicals or peroxides (CCl_3_ or CCl_3_OO) [130,131], via the cytochrome P450 pathway, thus causing lipid peroxidation and undermining the integrity of liver-cell membranes [132]. The administration of an SOD2 mimic (SOD2m) for 7 days has prevented the oxidative stress and inflammatory responses induced in the liver, by the exposure of mice to 0.05% CCl_4_, within 24 h. Indeed, a SOD2m-treated group showed a significant decrease in two crucial liver-injury biomarkers: aspartate aminotransferase (AST); and alanine aminotransferase (ALT). Accordingly, a reduction in histologically evaluated liver damage was observed. Moreover, the levels of several pro-inflammatory mediators, including prostaglandin E_2_ (PGE_2_), cyclooxygenase-2 (COX-2), interleukin (IL)-6 and tumor necrosis factor-*α* (TNF-*α*), were reduced [133].

The correlation between SOD and alcohol intoxication is now well established. Homozygous mutations in the SOD2 gene have been associated with a major risk of developing severe alcoholic liver disease in humans [134]. Interestingly, a study on a Han-Chinese population (80 patients with alcoholic cirrhosis, 80 patients with alcoholic non-cirrhosis, 80 with viral hepatitis B-related cirrhosis and 165 healthy controls) has demonstrated that patients with alcoholic cirrhosis had a higher frequency of the *SOD2 C/C* and *C/T* genotypes than the other groups, suggesting that the *SOD2 47T > C* genetic variant is a risk factor for alcoholic cirrhosis susceptibility [135]. On the other hand, moderate ethanol consumption (7–9 g/kg body wt/day) in SOD1^−/−^ mice promotes the onset and progression of alcoholic liver injury via a decrease in SOD2 and an increase in peroxynitrite contents, protein carbonyls and lipid peroxidation [136]. Accordingly, the adenovirus-mediated expression of SOD1 has been observed to be effective in reducing early alcohol-induced liver injury in rats [137]. More recently, SOD1 encapsulated in poly-L-lysine (PLL50)-polyethylene glycol (PEG) and then cross-linked with a reducible cross-linker (nano-SOD) reduced the steatohepatitis induced by ethanol in mice that were fed an ethanol liquid diet (5% of ethanol) for 4 weeks [138].

Several studies have associated a downregulation in SOD activity, and the consequent oxidative stress, with the progression of chronic skin damage induced by UV-irradiation [139]. SOD1 has been shown to exert a protective effect on human keratinocytes exposed to UVB [140]. Transfecting human keratinocytes with the SOD1 expression vector was effective in reducing UVB-induced apoptosis [141]. Moreover, a study on B16F10 murine melanoma cells has demonstrated that SOD1 (1–1000 ng/mL) inhibits melanin production within 24 h in a dose-dependent manner [142]. Accordingly, the topical administration of 1000 ng/mL SOD1 to HRM-2 melanin-possessing hairless mice before UVB 190 mJ/cm^2^ exposure decreased UVB-induced melanogenesis by blocking the aggravation of melanogenesis and thus potentially preventing melanoma development [142]. This evidence indicates the possible use of the exogenous supplementation or endogenous up-regulation of SOD to counteract UV-radiation-induced oxidative stress. An in vitro study demonstrated that the SOD mimetic belonging to the ethylenediamine chloride complex (EUK) family, EUK-134, increases human keratinocyte survival, after UVB-induced oxidative stress, via the indirect inhibition of the MAPK pathways [143]. Accordingly, the 30 U SOD/mL of the dried melon juice concentrate SODB, administered 24 h before UV exposure, has been seen to reduce keratinocyte apoptosis [139]. Moreover, the topical application of SOD, linked with the human immunodeficiency virus type 1 (HIV) transactivator of transcription (TAT) domain (TAT-SOD) at 300 U/cm^2^, 1 h before UVB irradiation, was effective in preventing UVB-induced erythema formation and blood-flow rise in Fitzpatrick skin type II and III subjects [144].

Similarly, it has been suggested that SOD2 is important in preventing the damage caused by UV radiation-induced oxidative stress, which can lead to numerous ocular pathologies [145]. Interestingly, an ophthalmic carbopol 934-based gel formulation, containing recombinant SOD2 (rMnSOD) as an active ingredient, reduced the number of microvilli damaged both in conjunctiva and cornea epithelial cells from rabbit eyes exposed to UV radiation [146]. The protective role of SOD in ocular damage may also have therapeutic implications in methanol intoxication. Visual symptoms usually occur within 12–36 h after ingestion and can be ascribed to the inhibition of cytochrome oxidase activity and the prevention of mitochondrial oxygen production in the optic nerve by formic acid, a toxic methanol metabolite [147]. Indeed, HCO_2_ can easily pass through the ganglion cell wall due to methanol-induced acidosis, leading to formate-oxidation reactions in the mitochondria and lysosome [148]. The optic nerve, retina and basal ganglia are the main tissues that are damaged by the increased oxidative-stress response [149]. The administration of tempol 2 h after methanol ingestion prevented the structural integrity of retinal ganglion cells in methanol-intoxicated rats [148]. Therefore, it is possible to hypothesize that SOD can be used as an antioxidant therapy for methanol-induced toxic optic neuropathy.

The ionizing radiation used in radiotherapy is known to trigger both ROS generation and the cytotoxic response, resulting in several different side effects, including fibrosis. When a deficiency in antioxidant enzymes is present, an increase in radio-sensitivity occurs [150]. The first observation of the beneficial effects of antioxidant therapy in preventing these events arrived in 1983, when a liposomal formulation of SOD was administered to two patients treated with high-dose pelvic radiotherapy, to reduce the fibrotic and necrosis response that occurred [151]. Since then, several publications have supported the role of SOD supplementation in radioprotection. The precise mechanisms responsible for the radioprotective effects of SOD are still unknown. Of the different possible forms of SOD, SOD2 is currently considered to be pivotal in protecting cells during exposure to ionizing radiation. Its importance has led to an investigation into the possible use of SOD activity in blood cells as a predictive biomarker for the selection of individualized irradiation therapy protocols. In an in vitro study of blood samples obtained from 32 breast-cancer patients, the activity of SOD after irradiation depended on initial SOD levels; these were decreased when initially high, and preserved when initially medium or low [152]. According to the authors, it is possible to consider patients with high basal levels of SOD to be poor responders, whereas patients with low basal levels may benefit from defense against the reactive free radicals produced after radiation. On the other hand, proton irradiation reduced SOD2 activity, while X-rays induced its overactivity [153]. This observation may be related to the bell-shaped dose-response curve observed following SOD administration. According to this, the optimization of concentration is essential in any application [9]. Therefore, SOD has been proposed as a strategy to prevent radiation-induced damage to different normal tissues. D. Leu and coll. (2017) have evaluated the effect of a lipophilic Mn porphyrin (MnP)-based SOD mimic, MnTnBuOE-2-PyP^5+^ (BMX-001), administered subcutaneously for one week before cranial irradiation and continued for one week afterward, in the radioprotection of hippocampal neurogenesis in a mouse model [154], and obtained promising results. Accordingly, MnTnHex-2-PyP^5+^, a similar SOD mimetic compound [155], delayed the onset of radiation-induced lung lesions, reduced respiratory-rate elevation and lessened the pathologic increases in lung weight in a model of radiation-induced lung injury in a non-human primate [156]. More recently, the MnP SOD mimetic AEOL 10150, also known as MnTDE-2-ImP^5+^, showed promising results in a whole thoracic lung irradiation model in nonhuman primates [157,158,159].

Moreover, the subcutaneous administration of bovine SOD (15 mg/kg) ameliorates radiation-induced lung injury in female rats by suppressing reactive oxygen species/reactive nitrogen species and ROS/RNS-dependent tissue damage [160]. Moreover, SOD3 administration has been tested in the treatment of radiation-induced pulmonary fibrosis. SOD3 has been recognized to be the main SOD form that is expressed in the lung, and is bound to the extracellular matrix [161]. The use of an association product that combines mesenchymal stromal cells (MSCs) with SOD3 was recognized as a promising strategy to counteract fibrotic processes: MSCs have already been reported to be effective in the early stages [162], but detrimental in the late stages [163] of pulmonary fibrosis, while SOD3 overexpression in the lung was recognized as being protective against the development of fibrosis [164]. The injection, 2 h post-irradiation, of SOD3-overexpressing MSC into mice that had been exposed to Cobalt-60 (20 Gy) was able to reduce collagen deposition, inhibit myofibroblast proliferation and reduce inflammatory cell infiltration,and consequently had an anti-fibrotic effect by preventing oxidative stress [165].

SOD had a generally beneficial effect on fibrotic response in a range of experimental settings. Melon-derived SOD has been administered in a gliadin oral formulation at 10,000 U/kg/day for 8 days to mice exposed to 25 Gy, 6 months before SOD treatment, and reduced the mean dermal thickness, which is predictive of radiation-induced fibrosis [166]. The same SOD formulation effectively reduced capsular fibrosis around silicone after implant surgery in an experimental model resembling breast-cancer treatment in rats [167]. However, the study failed to demonstrate that there was any beneficial effect in preventing or reducing radiation-induced fibrosis. These results are apparently in conflict with other previous studies that have had clear positive outcomes. However, the lower dose of SOD supplementation (500 mg/day for 3 weeks in the study [167] vs. 10,000 U/kg/day in the study [166]) and the use of different subcutaneously injected formulations [160], instead of oral administration, may account for these differences. The overall evidence for the use of SOD as a protective treatment in post-radiation fibrosis has led to at least two recently published clinical studies. However, the results obtained were not so comforting. The prospective study by K.C. Landeen and coll. (2018) [168] failed to demonstrate the effectiveness of topical SOD (280 U/g) at providing relief from the fibrosis of the head and neck area induced by radiation therapy in patients with a history of squamous cell carcinoma of the head and neck that had been treated with radiation. The study involved 68 adult patients, mostly males, and 86% had received radiation treatment at least 6 months before the initiation of the study. The improvement in the fibrosis score at 3 months was comparable in the SOD and placebo groups, suggesting that SOD had a marginal effect, compared to active physical therapy, in the post-treatment of neck fibrosis in patients with head and neck cancer [168]. Accordingly, the genetic association between SOD2 gene variations and radiation-induced soft-tissue toxicity has been reported in only one, monocentric, small-sample-size study [169]. On the other hand, a Phase 1b/2a study by C.M. Anderson and coll. (2018) [170] provided promising results regarding the effectiveness and safety of a cyclic polyamine SOD mimetic, avasopasem Mn or GC4419 (previously known as M40419, the enantiomer of M40403) at reducing the severe oral-mucositis that is induced by radiation-concurrent cisplatin in oral-cavity and oropharyngeal cancer. Patients (*n* = 46) with oral-cavity or oropharyngeal cancer, stages III–IVb, received fractionation intensity-modulated radiation therapy (once daily, Monday–Friday, at 2.0 to 2.2 Gy/d, to a cumulative tumor dose of between 60 and 72 Gy) with concurrent cisplatin (80–100 mg/m^2^ every 3 weeks or 30–40 mg/m^2^ weekly). GC4419 doses of 30 and 90 mg/day, administered throughout the chemoradiotherapy period, were the most effective and showed no particular safety concerns. These doses were therefore selected for the Phase 2b extension of the study [170].

5-fluorouracil is a chemotherapy agent known to cause severe mucositis and induce intestinal damage [171]. The administration of SOD was therefore also tested in a model of 5-fluorouracil-induced intestinal mucositis in mice. The study showed that Multi-modified Stable Anti-Oxidant Enzymes^®^ (MS-AOE^®^), an rMnSOD obtained from a mutant high-temperature-resistant SOD strain, alleviates the mucositis caused by 5-fluorouracil, primarily in the first 3–5 days [172].

Interestingly, oral mucositis is not the only side effect of cisplatin therapy that can be treated with SOD. SODs have also been proposed for the treatment of cisplatin nephrotoxicity. Indeed, cisplatin nephrotoxicity has been associated with ROS production, DNA fragmentation and the activation of caspase enzymes, especially caspase-3 [173,174]. The administration of tempol prevented a decline in the kidney function of rats that developed nephrotoxicity following a single i.p. injection of cisplatin 6 mg/kg [175]. Accordingly, rats treated with tempol showed an increase in kidney GSH content and SOD activity and a parallel decrease in kidney lipid peroxidation and NOx production [176].

Finally, a more recent example of SOD as a possible antidote has been proposed by Liu Z. and coll. (2020). The authors, using both an in vitro and an in vivo approach, demonstrated that bupivacaine induced the over-production of mitochondrial ROS, the activation of C-Jun *n*-terminal kinase (JNK), thus leading to SOD2 upregulation. On the other side, the SH-SY5Y cells transfected with SOD2 siRNA showed a higher susceptibility to bupivacaine, as demonstrated by the cell apoptosis increase. The SOD2 deletion induced mitochondrial ROS, malondialdehyde, and 8-hydroxydeoxyguanosine over-production, with a parallel decrease in the mitochondrial membrane potential. All these events were prevented by mito-tempo [177].

A summary of the proposed applications of SOD as a detoxification strategy, as discussed above, is provided in Table 1.

## 5. SOD as a Pharmacological Agent

The imbalance between oxidative-stress mediators and protective pathways, including SOD, has been recognized as a detrimental event in many pathophysiological disorders. This review highlights the most investigated applications of SOD as a therapeutic agent from 2012 to 2020, excluding the field of oncology (Table 2). Despite their differences in etiopathogenesis, oxidative stress has been recognized as a promoter of tissue damage. It can be argued that the generic antioxidant effects of SOD supplementation are beneficial in all of these conditions, from hypoxic damage and cardiovascular diseases to neurodegenerative disorders (Parkinson’s disease, Alzheimer’s disease, ALS), and metabolic diseases, including diabetes, its complications and obesity (Table 2).

However, it must be underlined that clinical evidence for this is limited, and consequently, real proof of efficacy is far from having been demonstrated. It is possible that the lack of clinical evidence of positive effects is, at least partially, due to the so-called “antioxidant paradox” [232], which is based on the cross-talk between oxidative stress and inflammation. These processes strictly influence each other and coexist in many pathological conditions. Therefore, a vicious circle is established: ROS and reactive nitrogen species (RNS) activate intracellular responses enhancing the expression of pro-inflammatory genes, and consequently, a number of pro-inflammatory mediators are released, and inflammatory cells are recruited. On the other hand, the inflammatory cells exaggerate the oxidative stress by producing ROS and RNS [232]. Several mediators participate in this vicious circle. Of these, a key role is played by the high-mobility group box protein 1 (HMGB1), a protein with a dual function: as a non-histone chromatin-binding protein involved in regulating transcription in the nucleus; and as a pro-inflammatory cytokine/chemokine when released into the extracellular space. Its relevance in oxidative stress-inflammation cross-talk is due to the extracellular form; ROS/RNS have been suggested to be both the cause and consequence of HMGB1 release [233]. Interestingly, a study on 86 patients with atrial fibrillation revealed a negative correlation between serum HMGB1 levels and SOD activity (r = −0.491, *p*  <  0.05) [234]. Moreover, HMGB1 translocation and release are promoted by H_2_O_2_ in hepatocytes [235], primary human epidermal melanocytes [236], and neonatal rat cardiomyocytes [237]. Therefore, the increase in SOD activity and the parallel reduction in HMGB1 levels have been proposed as the mechanisms underlying the protective effects exerted by quercetin in a rat model of sepsis [238], the amelioration of the cisplatin-induced hepatotoxicity by the *Ganoderma lucidum* mushroom [239], and the anti-inflammatory effect of the midazolam–sufentanil combination [240]. Two cross-talking pathways are involved: Nrf2/HO-1 and the Toll-like receptor (TLR)/NF-κB axis [241]. Indeed, HMGB1 can suppress the Nrf2 pathway [236,242], as well as activating TLR-4, and thus activates NF-κB signaling [243,244]. Considering its crucial role in SOD induction, the Nrf2 pathway is an attractive target for different chronic diseases in which oxidative stress is involved [245,246]. Therefore, pharmacologic modulators of Nrf2 may exert significant antioxidant effects through indirect SOD targeting, such as by PPAR activation. Nrf2-driven PPARγ induction was demonstrated to be protective against the pulmonary oxidant injury [247]. The review by I. Dovinova and coll. (2020) highlights PPARγ as one effector of SOD1, SOD2, and SOD3 expression in spontaneously hypertensive rats [248] and how this event contributes to pioglitazone’s therapeutic effects, including the control of blood pressure [249]. Moreover, S. Agarwal (2017) reviewed PPARs as promising therapeutic targets for several neurodegenerative disorders such as Parkinson’s, Alzheimer’s and Huntington’s disease, and ALS. In all these conditions, the role of oxidative stress has been recognized. Therefore, PPARs may have a beneficial effect even modulating SOD2 expression [250].

### 5.1. Ocular Diseases

In ophthalmology, oxidative stress is generically involved in ocular inflammation, and can thus contribute to the onset and progression of several eye diseases, including cataracts, age-related macular degeneration, uveitis, premature retinopathy, keratitis, glaucoma and dry-eye diseases [229,251].

Accordingly, SOD1 ocular instillation has been tested in several experimental models of uveitis, including allergic uveitis and acute corneal inflammation [230], and dry-eye disease [229]. In particular, the relevance of SOD in this disease has been underlined by the use of SOD1^−/−^ mice as an experimental model to test the benefits of several compounds on aqueous tear production [252]. Dry eye is a multifactorial age-associated disease, characterized by discomfort, visual disturbance and tear-film instability, that has the potential to damage the ocular surface [253]. SOD can have a dual influence on this disease; as a protective antioxidant and a detrimental pro-oxidant. A very recent cross-sectional study conducted on 51 patients that were affected by dry eye demonstrated a negative correlation, of −0.373, between the levels of SOD and the dry-eye degree. This negative correlation may be linked to a compensatory mechanism that occurs in the earliest phases [254]. The administration of SOD, or SOD mimetics, should be combined with an H_2_O_2_ scavenger to prevent further oxidative-stress propagation and prevent photoreceptor damage [255]. Interestingly, a case report, published in 2006 by L. Grumetto and coll., showed that the ophthalmic gel formulation of rMnSOD had protective effects in the treatment of bilateral posterior subcapsular cataracts [231].

### 5.2. Gastrointestinal Diseases

Oxidative stress contributes to various gastrointestinal diseases, such as gastroduodenal ulcers, inflammatory bowel disease (IBDs), and gastric colorectal cancer [256]. In particular, the rationale for SOD supplementation in gastrointestinal diseases stems from the observation that levels of SOD are relatively low in normal gut mucosa, and usually further reduced under inflammatory conditions [257]. For instance, enzyme levels are lower in Crohn’s-disease [258], and ulcerative-colitis patients [204]. However, in IBD patients, SOD levels are increased in the intestinal epithelial cells [259]. The higher SOD in IBD has been interpreted as a means of safeguarding intestinal tissues from oxidative damage. Accordingly, SOD levels in peripheral blood from IBD patients are increased, and they are currently used as a biomarker of oxidative stress. Moreover, SOD supplementation has been explored as a potentially beneficial strategy for preventing several different symptoms of bowel inflammation [260]. An experimental study by Y.H. Wang and coll. (2016) investigated the role of an SOD2m compound in a 2,4,6-trinitrobenzene sulfonic acid (TNBS)-induced colitis model in rats. This study demonstrated that 7-day treatment with the SOD2m compound elicited an antioxidant response that reduced colonic macroscopic and microscopic damage scores [207]. E. Mathieu and coll. (2017) obtained similar results by testing the cyclic polyamine SOD2m Mn1 in a mouse model of 2,4-dinitrobenzene sulfonic acid (DNBS)-induced colitis; Mn1 (4 mM/day via oral gavage for 7 days) slightly improved the macroscopic score of colitis [206].

Consistent positive effects have also been observed upon using a lecithinized Cu, Zn-SOD (PC-SOD) [204], a O-(2-hydroxyl) propyl-3-trimethyl ammonium chitosan chloride (O-HTCC) conjugated Cu, Zn-SOD (O-HTCC-SOD) [203], and a SOD2 that was recreated by a mutant high-SOD-producing *Bacillus amyloliquefaciens* strain [205], in a model of dextran sodium (DSS)-induced colitis in mice.

Both these experimental models of colitis, TNBS and DSS, cause severe inflammation with shortened, thickened and erythematous colons, as well as activating NF-κB and inducing the expression of TLR-4 and pro-inflammatory cytokines, such as IL-1β, IL-6 and TNF-α [261]. Therefore, we can argue that similar responses are activated regardless of the SOD form administered (Mn-SOD or Cu, Zn-SOD), and a reduction in the colonic inflammatory response is observed thanks to the downregulation of the TLR4/NF-κB signaling pathways [207].

### 5.3. Renal Diseases

SOD administration was promising when tested on the renal oxidative-stress response that occurs in chronic kidney disease (CKD), including diabetic nephropathy. In particular, a study by W. Ding and coll. (2015) has demonstrated the ability of tempol to improve renal function in a murine model of CKD that was surgically induced via 5/6 nephrectomy [219]. These data on tempol efficacy in CKD are consistent with those that demonstrate the benefits of tempol in cisplatin-induced nephrotoxicity [175]. Again, the SOD strategy was able to influence the pro-inflammatory response by downregulating the NF-κB signaling pathways. Moreover, a parallel downregulation of the pro-fibrotic response that is triggered by the TGF-ß/Smad-3 pathway was observed in the kidney [219]. Accordingly, administering tempol (1.5 mM/kg/day subcutaneously for 4 weeks) to diabetic rats has been observed to improve diabetes-induced glomerular injury, tubulointerstitial fibrosis and pro-inflammatory cytokine production [220]. Finally, tempol (1 mmol/L in drinking water for 5 weeks) prevented renal dysfunction in two-kidney, one-clip hypertensive rats. In particular, tempol prevented the development of hypertension, increased the plasma levels of urea, creatinine, and 8-isoprostane, preserved glomeruli number and kidney volume and prevented collagen deposition [221]. Consistent data have been obtained using spontaneously hypertensive rats in which tempol (1 mmol/L in drinking water for 8 weeks) increased SOD and nitric oxide synthases (NOS) activity in the kidney with a parallel reduction in NADPH activity and an additive effect to that of exercise (treadmill running for 20 m/min, 60 min/day, and 6 times/week) [222].

The anti-fibrotic effect exerted by tempol on the kidney was also exerted by human recombinant SOD3 (hEC-SOD) when chronically administered to diabetic rats [217]. hEC-SOD has therefore been proposed as a possible therapeutic agent to protect the progression of diabetic nephropathy in both Type 1 [217], and Type 2 [218], diabetes. These data also highlight the link between oxidative stress and the damage correlated with disturbed glucose homeostasis.

### 5.4. Metabolic Diseases

It is well known that SOD modulates metabolism; superoxide is generated from the metabolic processes that produce ATP from glucose and free fatty acids (FFAs), and SOD1 transgenic mice (G86R murine SOD1 mutation), which exhibit a gain-of-function mutation, are characterized by skeletal muscle hyper-metabolism, and a deficit in metabolism [262]. On the other hand, SOD1^−/−^ mice have shown worsened glucose homeostasis [263]. These data are consistent with the potential use of SOD as a metabolic regulator in a variety of diseases that are characterized by metabolic dysfunction, from insulin resistance to FFA accumulation and obesity. Obesity, in particular, is a strong independent predictor of systemic oxidative stress, as persistent obesity can deplete the source of the antioxidant [264]. Targeting SOD to improve their activity has been explored. In a high-fat diet model (20% protein, 35% carbohydrates and 45% fat, divided into 31.59% saturated, 35.51% monounsaturated and 32.91% polyunsaturated fatty acids for 8 weeks), obese mice were demonstrated to benefit from SOD supplementation with nano-SOD (1000 U/kg i.p. once every two days for 15 days). In particular, SOD administration reduced the levels of serum triglycerides [225]. The same formulation reduced the macrophage and inflammatory markers in visceral adipose tissue and the originating stromal cells [226]. These results were confirmed and strengthened by the same group in a more recent study, in which a combination of nonalcoholic steatohepatitis and alcohol-associated liver disease was experimentally obtained by feeding them a high-fat diet (45% fat calories diet) for 10 weeks before the chronic administration of ethanol (5% for 4 weeks). The treatment with nano-SOD (1000 U/kg i.p. once every two days for 15 days) was effective in attenuating the liver injury, improving adipose tissue lipid storage and reducing hepatic CYP2E1 [227].

Similarly, the MnP SOD mimetic, MnTE-2-PyP^5+^ (BMX-010, AEOL10113, 5 mg/kg subcutaneously every 3 days), has been shown to improve hepatic steatosis, the biomarkers of liver dysfunction, insulin sensitivity and glucose tolerance in a model of Type 2 diabetes that was induced by a high-fat diet (60% kcal fat for 12 weeks) [228]. Another study has made use of Golden Syrian hamsters that were fed a pro-obesity diet consisting of an excess of nine types of palatable industrially processed foods; highly fatty, sugary and salty, to induce obesity, insulin resistance and oxidative stress. In this model, 1-month SODB oral supplementation (10 U/day) decreased adipose tissue weight, oxidative stress and insulin resistance [223]. Interestingly, the same formulation prevented the effects of oxidative stress in another hamster model of obesity and insulin resistance that was induced by a high-fat diet [224]. The mechanism(s) underlying the metabolic role of SOD supplementation converge on transcriptional regulation and include: (i) an increase in SOD, GPx and CAT expression [223]; (ii) a reduction in the expression of genes that are involved in fatty-acid synthesis, as mediated by 5’ adenosine monophosphate-activated protein kinase (AMPK) signaling [225]; the oxidation of the NF-κB p50 subunit, thus impeding DNA-binding and transactivation [228,265].

### 5.5. Cardiovascular Diseases

Over time, a great deal of evidence has indicated that ROS reduction is an interesting cardiac-protection strategy [266,267]. The meta-analysis by W.C. Dornas and coll. (2015) has underlined the relevance of ROS in the pathogenesis of hypertension. Based on 28 out of 144 article studies on several different hypertensive animal models that were published between July 1998 and December 2012, tempol treatment has been demonstrated to be beneficial for mean arterial pressure [268]. Diminished SOD activity has been identified as a risk factor for stroke, hypertension, hypercholesterolemia, atherosclerosis, heart failure and other cardiovascular diseases [13], including coronary artery disease [269].

The most important link between ROS and hypertension is actuated through angiotensin II, the primary effector peptide of the renin-angiotensin system. Angiotensin II has been reported to increase intracellular ^•^O_2_^−^ levels following AT1 receptor activation on central neurons [270,271]. Accordingly, the intracerebroventricular injection of nano-SOD attenuated blood pressure in angiotensin II-dependent hypertensive mice [190]. Interestingly, the SOD melon extract SODB showed an inhibitory effect on the angiotensin-converting enzyme (ACE) in vitro [20]. In vivo, SODB has been observed to reduce the left ventricular weight index, cardiomyocyte size and stimulate endogenous antioxidant defense in a spontaneously hypertensive rat (SHR) model, in which the development and maintenance of hypertension, and its associated cardiac alterations, are underlined by oxidative stress. However, the reduction in blood pressure was only 5% (the comparator enalapril evoked a 20% reduction), thus suggesting that dietary supplementation with SODB during conventional antihypertensive therapy may be an interesting approach for cardiac hypertrophy [189]. Possible SOD efficacy in cardiovascular remodeling has led to SOD3 being recognized as maintaining extracellular matrix (ECM) homeostasis within the aorta media layer. Reduced levels of SOD3 have been localized in patients affected by ascending aortic aneurysms associated with the bicuspid aortic valve, and may thus contribute to the occurrence of ECM modifications [195]. Regarding the possible association between SOD3 polymorphism and cardiovascular risk, the debate is still open. A retrospective case-control study on 1470 blood samples collected in Khon Kaen Province, Thailand, between 2013 and 2017, from 735 control and 735 hypertensive subjects (mean age 59.3 ± 9.0 years) matched for age and sex demonstrated a tendency towards increased susceptibility to hypertension for the SOD3 rs2536512-GG genotype [272]. On the contrary, this variant was associated with a lower blood pression in a previous study on 1388 participants [273]. No association was found by X. Dong and coll. (2014) in a cohort of 343 hypertensive and 290 normotensive subjects [274].

Both endothelin (ET) system preservation [193], and an atheroprotective effect, via monocyte endothelial trafficking and transmigration suppression, can be counted among the various cardiovascular protective effects exerted by SOD agents [192].

Indeed, the MnP SOD mimetic AEOL 10150-injected s.c., reduced oxidative-stress markers, such as plasmatic isoprostane and 3-nitrotyrosine, as well as endothelins (ETs), in Fischer 344 rats, which are an inbred normotensive healthy rat model [193]. On the other hand, TAT-SOD, at 0.5 μM, inhibited the TNF-α-induced stimulation of vascular-cell adhesion molecule-1 (VCAM-1) in human umbilical vein endothelial cells (HUVECs), and integrin β1 in THP-1 monocytes. The prevention of transendothelial monocyte migration was supported by the firm localization of occludin-1, platelet/endothelial cell adhesion molecule-1 (PECAM-1), and vascular endothelial-cadherin at paracellular junctions, as well as the inhibition of endothelial matrix-degrading, matrix metalloproteinases (MMPs) [192]. The antioxidant effect of SOD at the cardiovascular level has also been demonstrated in human aortic endothelial cells (HAEC), in which nano-SOD decreased linoleic acid-induced oxidative stress, as demonstrated by the in vivo assessment of nano-SOD in vascular-cell activation in a mouse model of diet-induced obesity. Nano-SOD caused a significant decrease in vascular-cell activation in the thoracic aorta, in heart inflammation and in MMP expression in the aorta and ventricles [191].

Finally, a paper was published, in 2018, on SOD supplementation for the treatment of peripheral arterial disease (PAD). The study used the ligation of the femoral artery in rats as a model of PAD. This model causes an abnormal autonomic response that was significantly reduced after tempol administration [194].

### 5.6. Respiratory Diseases

Due to its specific functions, the respiratory apparatus is continuously and directly exposed to oxidative stress from the environment and pathogens. Moreover, it is exposed to higher oxygen tensions (∼13.3 kPa at the alveolus), and has a large surface area (adult human lungs: ∼140 m^2^) [275]. These anatomical features make the lung a unique organ, and one in which SOD is a primary defense from both the ROS produced during normal cell homeostasis, and the ROS produced as a consequence of lung diseases. ROS importance has been recognized in the etiopathogenesis of a variety of pulmonary diseases, including: asthma; chronic obstructive pulmonary disease; pulmonary fibrosis; asbestosis; cystic fibrosis; granulomatous lung disorders; sarcoidosis; allergic alveolitis; idiopathic interstitial pneumonia; primary pulmonary hypertension; and complications associated with lung transplantation [276].

In such a complex scenario, it is clear that SOD is an attractive strategy for the treatment of several pathologies. However, recent years have seen relatively few in-depth investigations, although pulmonary hypertension has probably received the most attention overall. Pulmonary hypertension is characterized by pulmonary vascular remodeling that leads to high blood pressure in the pulmonary artery and manifests as dyspnea both during exercise and at rest [277]. Therapy is currently based on a combinatorial approach of two or more drugs that are based on conventional vasodilators, but long-term outcomes are still suboptimal [278]. Exogenous SOD is a possible candidate for add-on therapy because of its radical scavenger activity, and its effect on the cardiovascular remodeling described above. The SOD mimetic, EUK-134, was therefore tested in a model of monocrotaline (MTC)-induced pulmonary hypertension in rats. In this study, EUK-134 (administered i.p. at 3 mg/kg/day for 4 weeks) prevented the force decrease and actin modification in the diaphragm bundles [201]. These results are in keeping with those obtained by L.R. Villegas and coll. (2013), who used another SOD mimetic, MnTE-2PyP^5+^. This compound attenuated chronic hypoxic pulmonary hypertension. More specifically, mice were exposed, for up to 35 days, to 10% atmospheric oxygen using a hypobaric chamber, and MnTE-2PyP^5+^ was administered s.c. at 5 mg/kg 3 times/week during the hypoxic exposure. The SOD mimetic proactive effect against the increased right ventricular systolic pressure and hypertrophy was sustained by a reduction in NLRP3 (nucleotide-binding domain leucine-rich repeat (NLR) and pyrin domain containing receptor 3) inflammasome activation [202].

Finally, N. Gupta and coll. (2017) have formulated an inhalable combination therapy, consisting of the vasodilator fasudil and SOD1, which was formulated in liposomes equipped with CARSKNKDC (CAR), which is used as a homing peptide. The drug has been tested in rats in both MTC-induced acute pulmonary hypertension and Sugen 5416 hypoxia-induced chronic pulmonary hypertension models. In the acute model, the CAR-modified liposomes that contained fasudil and SOD elicited a more pronounced, prolonged and selective reduction in the mean pulmonary arterial pressure than the unmodified liposomes and plain drugs. In the chronic model, the effect induced by the CAR-modified liposomes containing fasudil and SOD reduced the mean pulmonary arterial pressure by 50% and slowed the right ventricular hypertrophy [196]. The obtained results therefore support the possible use of SOD as an add-on therapy in pulmonary hypertension.

Ischemia/reperfusion of the lung is usually associated with the unilateral-lung transplantation that is required when end-stage respiratory failure occurs. The occurrence of pulmonary ischemia/reperfusion inevitably causes the massive production and release of superoxide radicals and inflammatory cytokines [279], with MMP activation [280]. Therefore, it is not surprising, considering the homology with observations at the cardiovascular level, that SOD1 (1000 U/kg i.v.) has been shown to attenuate ischemia/reperfusion-induced contralateral lung injury by reducing pulmonary permeability, lipid peroxidation and MMP activity [198].

SOD has also been tested as a protective agent during mechanical ventilation. Indeed, the overinflating of the alveoli and repeated stretching of lung tissues promotes redox imbalance and inflammatory responses [281]. It has been recognized that the detrimental events that occur during this mechanism can be treated with an antioxidant strategy, such as an SOD-based therapy. For example, PC-SOD suppressed induced lung injury, improving lung edema and elastance, in an experimental mechanical ventilation model [197]. Furthermore, SOD1, administered at 1000 U/kg/h i.v. to rats that underwent 5 h ventilation with a high tidal volume (18 mL/kg), preserved lung-function integrity by reducing both pulmonary oxidative stress and inflammation, preserving pulmonary-surfactant expression and enhancing vascular NO bioavailability [199].

Lung protection during sepsis is another context in which SOD has been tested. The inflammatory response in sepsis triggers ROS production in the lung [282]. Therefore, SOD treatment may be effective for lung protection in this case as well. A paper by L. Constantino and coll. (2014) demonstrated that the metal-based SOD mimetic [Fe(HPClNOL)Cl_2_]NO_3_ decreases nitrotyrosine and pro-inflammatory cytokine and improves lung permeability in septic rats [200].

### 5.7. Neurological Diseases

The central nervous system is very sensitive to oxidative stress, with regions such as the prefrontal cortex, the hippocampus and the amygdala being particularly susceptible to oxidative-stress-related functional decline [283]. The consequent damage can lead to neurodegenerative disorders that are associated with muscular and cognitive deficits, dementia and psychiatric disorders. Indeed, oxidative stress has been reported to have a detrimental effect on the formation of neuronal plaques, the amyloid β protein in Alzheimer’s disease, α-synuclein in Parkinson’s disease and the mutant Huntington protein in Huntington’s disease [284]. Simultaneously, oxidative stress is also involved in some psychiatric disorders, including depression, anxiety, schizophrenia and the autism spectrum [285]. On this basis, using antioxidants as a pharmacological strategy for a broad spectrum of neurological applications has been hypothesized. Despite these assumptions, a relatively low number of papers have explored the role of SOD as a therapeutic intervention. One of these is a randomized, double-blind, placebo-controlled clinical pilot study investigating the use of 12-week-long SODB supplementation (Extramel^®^ 140 U of SOD, Bionov, Eyragues, France) on psychological stress, and physical and mental fatigue in 61 healthy volunteers. Supplementation was effective against perceived stress and fatigue [178]. Similar results have recently been reported in a monocentric, controlled trial vs. the placebo, randomized, double-blind trial performed from November 2016 to March 2018. The study included 41 healthy volunteers (all men, mean age of 38.8 years old, body mass index between 18.5 and 29.5 kg/m^2^) with a stable weight and a stable diet over the past 3 months and no contraindication to the practice of running. The study demonstrated a lower initial inflammatory state in the SODB group which was maintained during and after the training session, whereas the placebo group experienced a significant increase in inflammation. The authors identified an increase in the PARγ coactivator 1-alpha (PGC-1alpha) and the consequent myosin fibers rearrangement the lading pathway for adaptation to effort, endurance, performance [179].

Other applications have only been investigated at the experimental level. *S. cerevisiae* is a suitable eukaryotic model for aging as it recapitulates the susceptibility of human cells to the proteotoxicity of α-synuclein, amyloid-β, the poliQ trait of Huntington’s and mutant forms of SOD1 [180]. This model has been used to investigate the possible use of SOD mimetics as therapeutic agents against aging-related diseases, such as Parkinson’s and Alzheimer’s, and promising results have been obtained [286].

A study by A. Clausen and coll. (2012) has investigated the effect of the SOD/CAT mimetic EUK-207 on learning and memory in an experimental model of Alzheimer’s disease. The compound, which had already been tested on age-related learning and memory impairment in mice [184], was administered to triple-transgenic Alzheimer’s disease (3xTg-AD) mice that expressed mutant forms of the amyloid-protein precursor and presenilin 1 (found in hereditary forms of Alzheimer’s disease), and a mutated form of the microtubule-associated protein tau (associated with frontal temporal dementia) [287]. EUK-207-treated 3xTg-AD mice did not display any deficit in fear conditioning while, in parallel, reduced tau and phosphorylated tau accumulation were observed in the amygdala and hippocampus and reduced nucleic acid oxidation and lipid peroxidation were observed in the brain [185]. Using the TgCRND8 Alzheimer’s disease model, which is a transgenic mouse model that presents an aberrant cleavage of the amyloid β precursor, it has been demonstrated that oral SOD supplementation reduces thiol levels in plasma [181].

SOD mimetics have also been investigated as a potential treatment for stroke. This is not surprising when we consider that increased ROS levels cause protein, lipid and DNA damage after cerebral ischemia. Accordingly, the neuroprotective effect of the MnP SOD2m MnTM-4-PyP^5+^ has been demonstrated in a mouse model of the transient occlusion of the middle cerebral artery (MCAO). The study showed a reduction in infarct volume and improved neurological function after the intravenous administration of MnTM-4-PyP^5+^, 30 min before surgery [186]. Similar effects were observed in rats that were subjected to MCAO in order to investigate tempol microdialysation (10 mM) and intracerebroventricular injection (500 nmol 15 min before MCAO). The functional benefits observed were sustained by reducing glutamate, aspartate, taurine and alanine release [187].

As described above, the association between SOD and ALS, mostly highlighted in the KO studies, is also very interesting. However, it has yet to be established whether a loss of function is the underlying mechanism in SOD1-related motor neuron disease, meaning that the usefulness of SOD targeting as an approach for ALS has yet to be defined [288,289,290].

Interestingly, the results of a first clinical study (Phase 1/2 trial) to test the efficacy of tofersen, an antisense oligonucleotide that mediates the degradation of the SOD1 messenger RNA to reduce SOD1 protein synthesis in ALS patients, have just been published in the New England Journal of Medicine [291]. The results are promising, and tofersen is already undergoing a Phase 3, randomized, double-blind, placebo-controlled trial with long-term extension included (ClinicalTrials.gov numbers, NCT02623699 and NCT03070119, respectively, accessed on 19 March 2021).

The increased oxidative stress status has also been recognized in Down syndrome. This syndrome is due to the trisomy of chromosome 21. Therefore, the overexpression of genes located on chromosome 21 (including SOD1) is considered to be an essential feature for the Down syndrome phenotype [292]. Several reports have demonstrated the overexpression and/or overactivation of SOD1 not only in the amniotic fluid of Down syndrome fetuses [293], but also in several cells and tissues of Down syndrome patients. For instance, N.B. Domingues and coll. (2017) have demonstrated that SOD activity is increased in the saliva of children with Down syndrome compared to the control group [294]. Similar results were obtained in cultured primary nasal epithelial cells from Down syndrome children that exhibited an increased in SOD1 content (about 28%), compared to children with a normal karyogram [295], as well as in the plasma of Down syndrome children [296]. The cognitive impairments and premature signs of aging associated with Down syndrome have been associated with the SOD1/GPx ratio in the brain [297]. Despite all this evidence, antioxidant supplementation was not effective on the cognitive functions of Down syndrome patients [298]. Therefore, SOD targeting is currently not a recommended strategy [299], and further studies evaluating a variety of SOD supplements, dose-escalation and the duration of administration should be considered.

As mentioned above, neural tissue is particularly susceptible to ROS damage, and ROS accumulation in the spinal cord is considered crucial in the development of neuropathic pain [300]. SOD has consequently been tested in a chronic model of central pain that was induced by spinal cord injury (L1 spinal contusion in rats). SOD was i.p. administered and able to increase the paw-withdrawal threshold, thus indicating that there was a reduction in mechanical allodynia. The enhancement of spinal phosphorylated NMDA receptor subunit 1 (pNR-1) has been indicated as a possible mechanistic interpretation of this effect [182]. Similar results were also obtained in another model of neuropathic pain. Unilateral painful C7 root compression, where free SOD was compared with a different form of SOD preparation, was performed in rats; the SOD-loaded porous polymersomes were more effective than free SOD because of their better bioavailability [183].

The effect of SOD on inflammatory pain has also been tested. For instance, in a model of potassium superoxide (KO_2_)-induced pain and inflammation in mice, tempol (10–100 mg/kg) was i.p. injected 40 min before the intraplantar injection of KO_2_ and was able to reduce mechanical and thermal hyperalgesia and paw edema. Tempol has also been observed to have similar beneficial effects in both carrageenan and complete Freund’s adjuvant inflammatory hyperalgesia models. The mechanisms underlying the analgesic and anti-inflammatory effects involve the inhibition of the glial markers that are induced in the spinal cord, and an increase in Nrf2, which is downregulated by KO_2_ injection into paw skin and the spinal cord [188].

### 5.8. Skin Diseases

The skin barrier is a primary defense system that protects the body from harmful external insults, making oxidative stress and the consequent production and accumulation of ROS critical. In wound healing, ROS participates in the inflammatory phase, during which a variety of immune cells are recruited, and ROS are generated, in large amounts, to counteract invading pathogens and promote their phagocytosis. However, the downside is the overproduction of superoxide and peroxynitrite, which can negatively affect the surrounding tissues [301]. The role of SOD as a radical scavenger appears to be clear in this setting, and its use in wound repair is attractive. Accordingly, an SOD1-based hydrogel of carboxymethylcellulose has been observed to improve the healing of open wounds on the back skin of rats by stimulating fibroblast proliferation [208]. Consistently, a novel SOD-loaded thermo-sensitive hydrogel-poly(N-isopropyl-acrylamide)/poly(γ-glutamic acid) was developed by Y. Dong and coll. (2020). This formulation showed good biocompatibility and a wound closure rate after 21 days of operation, of up to 92% in diabetic rats [213]. Furthermore, SOD2 stimulated wound healing in streptozotocin-induced type I diabetes rats [209]. The efficacy of a strategy that combines SOD2m MnTE-2PyP^5+^ and negative pressure wound therapy (NPWT), a widely used management tool in surgical and trauma wounds, has more recently been investigated. The preclinical study demonstrated that MnTE-2PyP^5+^ is a wound-healing enhancer; its topical application promoted wound closure within two days [212]. A similar approach, which uses the properties of SOD to enhance the therapeutic effects of other therapies, involves the formulation of MSC that overexpress SOD3. This treatment has been tested in both psoriasis [214], and dermatitis [215]. In this approach, the immune-modulatory effects of MSCs are enhanced by the antioxidant effect of SOD3, which also shows anti-inflammatory properties. MSCs have long been studied for their properties and importance in managing several skin diseases, including: wound healing; burn injuries; epidermolysis bullosa; systemic lupus erythematosus; dermatomyositis; systemic sclerosis; photoaging; acne; psoriasis; and atopic dermatitis [302]. SOD3-overexpressing MSCs specifically prevented the development of psoriasis in a mouse model of imiquimod (IMQ)-induced psoriasis-like inflammation via the inhibition of the TLR7/MAPKs/NF-κB axis and the activation of the adenosine receptor [214]. Similarly, SOD3 inhibited TLR2/MAPKs/NF-κB and the NLRP3 inflammasome, and consequently suppressed inflammation in a mouse model of *Propionibacterium acnes*-induced skin inflammation [210]. Moreover, SOD3 suppressed the inflammatory response induced in human keratinocytes and mast cells by cathelicidin (LL-37) and serine protease kallikrein-5 exposure (KLK-5), suppressing the activation of epidermal growth factor receptor (EGFR) and the p38 MAPK pathway [211].

The immune-modulatory and anti-inflammatory effects of MSCs that overexpress SOD3 also proceed via the inhibition of histamine H_4_ receptor expression and consequently, of the associated signaling cascade in murine dermatitis-like skin inflammation, as induced by ovalbumin [215]. Consistently with the demonstration of SOD as a therapeutic agent for skin diseases, the most recently published data explore a new SOD mimetic, the RM191A: a water-soluble dimeric copper (Cu^2+^-Cu^3+^)-centered polyglycine coordination complex with superoxide quenching activity 10-fold higher than that of SOD. This compound, which is under Phase 2 investigation for the relief of neuropathic pain as a local spray (registration number ACTRN12617000206325; https://www.anzctr.org.au, last accessed on 19 March 2021), was demonstrated to accelerate excisional wound healing, reduce 12-*O*-tetradecanoylphorbol-13-acetate (TPA)-induced inflammation, and attenuate age-associated oxidative stress in skin when administered to mice as topical gel [216].

## 6. SOD Sources

Different SOD-based compounds have been tested; from plant and animal extracts and SOD recombinant forms to SOD mimetics and SOD gene therapy (Table 3). 

This heterogeneity stems from the need for an exogenous SOD with optimal pharmacokinetics properties. Exogenous SOD has relatively low bioavailability, especially when orally administered. Indeed, due to its enzymatic nature, exogenous SOD is digested and denatured in the stomach. Moreover, it should be noted that exogenous SOD has a high molecular weight, meaning that cellular uptake is limited, even when it is injected [21]. These aspects explain why SOD use is restricted to drug applications in animals, and to non-drug applications in humans (including supplements, cosmetics, food, agriculture and chemical industries) [303]. Although exogenous SOD administration has often proven problematic, a variety of innovative approaches are currently being explored [12]. SODB has been considered the gold standard for the dietary supplementation of SOD since 2000. However, its efficacy is affected by the low pH and high proteolytic activity in the digestive tract [16]. Research on designing formulations with SOD encapsulated in lipids and/or proteins has been performed to overcome the low bioavailability of natural SOD. Thus far, gliadin-SOD, nano-SOD and O-HTCC-SOD (Table 3) have been created. These products should protect the enzyme from degradation, but do not entirely solve the absorption problem caused by SOD’s high molecular weight [16]. Indeed, if the formulation scaffold cannot activate tight-junction promoting absorption, intestinal permeability is still limited. Therefore, another strategy has been pursued since the late 1970s; the development of synthetic antioxidant enzymes, SOD mimetics, that were developed to overcome the bioavailability problem of SOD supplementation. SOD mimetics are characterized by low-molecular weight (about 483 Da) and better intestinal permeability when administered orally, but this also grants a higher circulating half-life and lower antigenicity [24]. Approaches for the future of this field seem to include gene therapy to produce more antioxidants in the body, for instance, by creating stem cells that overexpress the SOD enzyme via genetic modifications. The development of SOD3-overexpressing MSCs is being investigated in this field. The aim here is to overcome the limits of MSC therapy, such as circumscribed survival and reduced immunomodulatory potential, using the benefits of SOD3 antioxidant and immunomodulatory activity [304]. Future studies will provide more in-depth knowledge of the feasibility of this strategy. In addition to these pharmaceutical approaches, several sources of exogenous SOD have been pursued. SOD was formerly obtained from the liver and serum of mammals such as pigs, horses, bulls and dogs [303]. Of these, bovine-derived SOD, known as orgotein, has been licensed as a veterinary product for use as a non-steroidal anti-inflammatory drug (ATC code M01AX14). Nowadays, if not of human origin, (recombinant human SOD), SOD is mostly derived from terrestrial and marine plants, microbial, cyanobacterial and chromista sources (Table 4). However, marine and terrestrial fungi, as well as yeasts, are also important sources of SOD.

The use of SODs from various sources reflects the need to emphasize different properties of different forms of the enzyme, with the different sources mainly used for specific applications; plant-derived SOD is mostly used for supplements and nutraceuticals, while SOD from marine source is used in cosmetics [305]. Plants have three isoforms of SOD: chloroplastic and cytosolic Cu, Zn-SOD; mitochondria Mn-SOD; and chloroplastic and plastidial Fe-SOD [21,306]. Cyanobacteria and marine creatures contain the Ni-SOD isoform. Cu, Zn-SOD, Ni-SOD and Fe-SOD are very sensitive to H_2_O_2_, but Cu, Zn-SOD and Ni-SOD are also sensitive to cyanide. Mn-SOD is insensitive to both H_2_O_2_ and cyanide [306]. Fe-SOD has also been found in prokaryotes, including marine bacteria, such as *Photobacterium leiognathi* and *Photobacterium sepia*, as well as in protozoans and the chloroplasts of algae such as *Lingulodinium polyedrum* [303]. The use of these SODs for large scale commercialization has often been limited by the minimal SOD content and the high cost of extraction methods. Therefore, the most used source of SOD has been *Cucumis melo L.C*., in which SOD is extracted from dried melon pulp with relatively high efficiency; 1 kg of freeze-dried concentrated melon juice [307], containing 90,000 U/g of SOD, is obtained from 15 kg of melon pulp, after filtration and concentration steps [20]. By comparison, other terrestrial plant sources of SOD have a low abundance of the enzyme. For instance, it is possible to extract 5–44 U/min/g fresh weight of SOD from sugarcane leaves [21]. However, several strategies have been developed to enhance SOD activity, more than 100-fold, and make these alternative sources effective drugs. For instance, Z. Hou and coll. (2019) have significantly improved the extraction of SOD from sea buckthorn and chestnut rose by adding purification steps, such as ammonium sulfate precipitation and anion exchange chromatography [308]. Furthermore, changing the salinity or adding heavy metals to the ground has been seen to provoke water deficiency, as does reducing or increasing the temperature, inducing an oxidative-stress response that led to SOD-content increases [21]. Moreover, culinary herbs such as *Rosmarinus officinalis*, *Thymus officinalis* and *Salvia officinalis* possess SOD mimetic activity that can even increase when cooked, or cooked and digested [309]. 

According to the literature sources reported herein, SOD mimetics can be divided into different classes according to their structure: cyclic polyamines; MnPLEDs; MnP; salen–Mn complexes; three metal-based compounds; and nitroxides (Table 5).

These structural differences can result in differing pharmacokinetic properties, including the route of administration and subsequent bioavailability. While the pharmacokinetics of MnPs have been widely investigated, a similar in-depth pharmacokinetic analysis is not available for other SOD mimetics, as reported by I. Batinic-Haberle and coll. (2018) [25]. Therefore, a proper pharmacokinetic comparison of the different SOD-based strategies is not possible.

Useful tools for classification can be found in the review by R. Bonetta (2018) [24]. A more extensive analysis of MnP compounds has been reported by I. Batinic-Haberle and coll. (2018) [25], I. Batinic-Haberle and I. Spasojevic [26] and I. Batinic-Haberle and M.E. Tome [27]. Briefly, the cyclic polyamine class differs in the metal, Fe^2+^, Mn^2+^ and even Cu^2+^, as well as in the polyamine moiety. However, they all have a dose-proportional response curve [310,311], instead of a bell-shaped dose-response curve, which is characteristic of the natural SOD enzyme [312]. Within this class, GC4419 has been extensively described. Developed by Galera Therapeutics, Inc. as a 1,4,7,10,13-pentazazcyclopentadecane derivative, it has already been tested in humans for the treatment of oral-mucositis as induced by radiation-concurrent cisplatin treatment [170]. MnPLEDs have several antioxidant properties, including the inhibition of SOD, GPx and CAT activity, as well as iron-binding, and consequently, the Fenton reaction. Accordingly, these compounds inhibit the formation of both ONOO^−^ and ^•^OH and increase H_2_O_2_ detoxification [313]. MnP-based SOD mimetics combine the effect evoked by the Mn moiety on ^•^O_2_^−^ dismutation, via the reduction of Mn^3+^ to Mn^2+^ and its oxidization back to Mn^3+^, with the CAT activity that is attributed to the porphyrin radical cation’s ability to undergo oxidation to higher oxidation states, Mn^4+^ or Mn^5+^ [314]. Moreover, MnPs can produce H_2_O_2_ by first undergoing rapid one-electron reduction with endogenous or exogenous ascorbate or thiols, and then being re-oxidized by O_2_ or ^•^O_2_^−^ [25]. MnPs offer several favorable features, including the absence of antigenicity, high stability that assures the integrity of the metal site, and low molecular weight [315]. Moreover, various delivery systems can reduce side effects such as acute hypotensive response observed with MnTnBuOE-2-PyP^5+^. Under this task, S.L. Schlichte (2020) developed a mesoporous silica nanoparticle and lipid bilayer nanoformulation of MnTnBuOE-2-PyP^5+^. The nanoformulation allows a slow and sustained release of the drug, thus reducing the acute reduction in renal sympathetic nerve activity induced by the injection of the free drug [316].

From a mechanistic point of view, this class is far beyond just being a radical scavenger as they add the reaction with H_2_O_2_, ^•^O_2_^−^ and ONOO^−^ to that with thiols. This last property is responsible for activating the Keap1/Nrf2 pathway, which is responsible for transcriptional activity, and SOD upregulation [25,27]. Salen-Mn complexes are thought to have SOD/CAT biomimetic activity. A multi-step process describes their mechanism of action: (i) interaction with ^•^O_2_^−^ reduces Mn^3+^ to Mn^2+^; (ii) Mn^2+^ is oxidized back to Mn^3+^ by ^•^O_2_^−^ consumption; (iii) salen-manganese is oxidized to salen-oxomanganese by H_2_O_2_; (iv) salen-oxomanganese is then reduced to salen-manganese by H_2_O_2_, liberating H_2_O and O_2_. Moreover, they have even been reported to scavenge RNS. The EUK compounds belong to this class. In particular, EUK-134 is a first-generation compound (it has a non-cyclized structure), while EUK-207 is a second-generation compound with greater stability due to this cyclized structure [24,317]. More recently, another class of SOD/CAT mimetics has been added; the metal-based compounds. This class has a conserved core, 1-[bis(pyridin-2-ylmethyl) amino]-3-chloropropan-2-ol (HPClNOL), that can be complexed with Fe^3+^, Mn^2+^ and even Cu^2+^. Collectively, this class possesses intrinsic ^•^O_2_^-^ and H_2_O_2_ scavenger activity. Compared to the salen–manganese complexes, the metal-based compounds have not been observed to affect the capacity of cells to synthesize neutral lipids and to compartmentalize them into lipid droplets. Cell-membrane integrity is thus maintained, hinting at possible higher efficacy against aging [286]. However, further studies that explore the real mechanism of action of these compounds must be performed to support this hypothesis. The classification of nitroxides as SOD mimetics is more controversial. Some authors, like S. Miriyala and coll. (2012), have highlighted the inability of nitroxides to catalytically scavenge superoxide [126]. Their mechanism includes reducing hydroxylamine within mitochondria [315], where these compounds display a weak and pH-dependent SOD-like activity [24]. According to this mechanism, some authors include nitroxide among SOD-mimetic compounds. However, this remains a controversial proposal [24,176,188,220,318]. The discussion includes the classification of tempol and mito-tempo as SOD mimetics. Tempol acts as a redox-cycling nitroxide water-soluble SOD mimetic [24,176,188,220,318], and shares the activation of the PI3K/Akt/Nrf2 pathway with other SOD mimetics [124,319,320]. Accordingly, the combination of tempol with the TPP+ moiety, resulting in mito-tempo, is accepted as a SOD mimetic [127,321]. According to the mechanistic interpretation of the SOD mimetic based on their ability to activate the PI3K/Akt/Nrf2 pathway, other inducers of this pathway can be included among the “source of SODs”. Therefore, the following could be added to the list: (i) the several NRF2 activators such as dimethyl fumarate, bardoxolone methyl, sulforaphane, curcumin, quercetin, and metformin; (ii) the PPARγ activators such as the antidiabetic drugs glitazones, ankaflavin, monascin, and carotenoids; or (iii) the dual Nrf2 and PPARγ activators, genistin, olmesartan, 18β-Glycyrrhetinic acid, and resveratrol, included [47,48,245].

## 7. Conclusions

The literature data that have been reported herein, covering papers published between 2012 and 2020 on the use of SODs for neurological, cardiovascular, respiratory, gastrointestinal, renal, skin, metabolic and ocular diseases, are indicative of the high efficacy of all the SOD types tested, both natural SOD and SOD mimetics. Although SOD has been an attractive potential therapeutic approach for 50 years, most of the published papers, and even more so in the case of recent works, deal with experimental preclinical studies, and only comparatively few clinical studies are ongoing. Notably, the spread of the pandemic COVID-19 infection, causing the severe acute respiratory syndrome coronavirus 2 (SARS-CoV-2), further renewed the interest in pharmacological strategies to counteract the oxidative stress response triggered by NOS. Accordingly, J.O.C. Karlsson and coll. (2020) proposed mangafodipir to lower the inflammatory burden in critical SARS-CoV-2 infections [322]. In addition, just in September 2020, Galera Therapeutics, Inc. announced the first randomized, double-blind pilot phase II clinical trial with GC4419 for COVID-19 (ClinicalTrials.gov numbers, NCT04555096, accessed on 19 March 2021). However, none of the tested compounds have been approved to date. Several issues with the testing conditions and the type of compound evaluated have hampered the translation of the evidence for SOD use from the bench to the bedside. These topics can be summarized in three major points.

Firstly, the heterogeneity of the various compounds used to enhance the levels of SOD, from SOD extracts and SOD recombinant forms to SOD mimetics and SOD gene therapy, is an issue, as is the lack of comparative head-to-head studies. This point is strictly correlated with the second, which is the problem of bioavailability and the route of administration for effective doses in humans, and the timing of administration in relation to the dynamics of pathological process. Indeed, the optimal conditions for all the therapeutic approaches have not yet been clearly established. In the absence of comparative studies, even pharmacokinetics and toxicology data are not sufficient for a conclusive consensus on which sources of SOD, doses and administration timings best reflect clinical needs. This is also true for SOD mimetics, which are the most extensively studied type. MnPs are the only compounds for which pharmacokinetics have been clearly defined [25]. However, there is a lack of comparative studies against other sources of SOD and gold-standard comparators—even here.

The third issue is the heterogeneity of the diseases in which SOD strategies have been tested. Indeed, different compounds have been tested for similar applications, but have not been compared. Furthermore, although the same compound has been used under different pathological conditions, reported data cannot still define a specific indication for human use. The spectrum of diseases evaluated is vast, and detrimental contributions by ROS have been comprehensively demonstrated in each. However, oxidative stress can be considered a generic mechanism present in almost all pathological processes, and it is not unique to pathophysiological contexts. Therefore, its role as a drug target may vary according to the disease type and underlying biochemical processes. The way in which SOD affects oxidative stress may be regarded as a composite of direct (scavenger activity) and indirect (stimulating gene transcription of antioxidant pathways) antioxidant effects, as previously discussed.

The role of SOD merits a different type of discussion when considering ALS, in which a mutant overactive SOD1 has been identified, and Down syndrome, in which chromosome 21 trisomy has been associated with the overexpression of SOD1 in patients. These last two diseases remind us that SOD is a hormetic substance; added or over-expressed SOD produces potential beneficial effects in almost all of the conditions tested. However, in some circumstances, the benefits of SOD are either not so clear (i.e., gastrointestinal diseases) or even detrimental (i.e., ALS), in that they can exacerbate cell injury and death [284]. The interpretation of SOD as a hormetic substance draws our attention to another adjunctive issue when defining the therapeutic potential of SODs—the selection of the dose for optimal and tight regulation.

These issues collectively confirm the role of SOD as a supplement, but do not yet allow SOD to be conclusively repositioned as a drug that can be applied in the real world. Further evidence from the ongoing clinical trials is eagerly anticipated.

## Figures and Tables

**Table 1:** Possible applications of SODs as a detoxification strategy.

Insult	Treatment Tested	Reference(s)
paracetamol hepatotoxicity	mangafodipir	[125]
	mito-tempo	[127]
	tempol	[124]
	calmangafodipir	[128] *, [129]
carbon tetrachloride intoxication	SOD2m	[133]
alcohol intoxication	SOD1	[137]
	nano-SOD	[138]
methanol intoxication	tempol	[148]
UV-induced skin damage	SODB	[139]
	SOD1	[140,141,142]
	TAT-SOD	[144]
	EUK-134	[143]
UV-induced ocular pathologies	rMnSOD	[146]
radiotherapy-induced cytotoxic response	gliadin SOD	[166,167]
	SOD	[160], [168] *
	SOD3	[165]
	GC4419	[170] *
	MnTnBuOE-2-PyP^5+^	[154]
	MnTDE-2-ImP^5+^	[157,158,159]
	MnTnHex-2-PyP^5+^	[155,156]
	SOD3-overexpressing MSCs	[164]
cisplatin-induced oral mucositis	GC4419	[170] *
cisplatin-induced nephrotoxicity	tempol	[175]
5-fluorouracil-induced intestinal mucositis	MS-AOE^®^	[172]
Bupivacaine-induced neurotoxicity	mito-tempo	[177]

MS-AOE^®^ = Multi-modified Stable Anti-Oxidant Enzymes^®^. SODB = Cucumis melo L.C. derived SOD, nano-SOD = SOD1 encapsulated in poly-L-lysine (PLL50)-polyethylene glycol (PEG), cross-linked with a reducible cross-linker, TAT = human immunodeficiency virus type 1 (HIV) transactivator of transcription, SOD2m = SOD2 mimetic, MSC = mesenchymal stromal cells, * clinical study.

**Table 2:** Potential SOD applications tested in animal models of human disease and clinical trials between 2012 and 2020.

Application	SOD Formulation	References
neurological diseases	SODB	[178] *, [179] *
	SOD1	[180]
	SOD	[181,182]
	SOD-loaded porous polymersome	[183]
	EUK-207	[184,185]
	MnTM-4-PyP^5+^	[186]
	tempol	[187,188]
cardiovascular diseases	SODB	[20,189]
	nano-SOD	[190,191]
	TAT-SOD	[192]
	MnTDE-2-ImP^5+^	[193]
	tempol	[194]
	SOD3-overexpressing MSCs	[195]
respiratory diseases	CAR-modified liposomes fasudil plus SOD	[196]
	PC-SOD	[197]
	SOD1	[198,199]
	[Fe(HPClNOL)Cl_2_]NO_3_	[200]
	EUK-134	[201]
	MnTE-2-PyP^5+^	[202]
gastrointestinal diseases	O-HTCC-SOD	[203]
	PC-SOD	[204]
	SOD2 by *Bacillus amyloliquefaciens* strain	[205]
	Mn1	[206]
	SOD2m	[207]
skin diseases	SOD1	[208]
	SOD2	[209]
	SOD3	[210,211]
	MnTE-2-PyP^5+^	[212]
	SOD-loaded thermo-sensitive hydrogel-poly(*N*-isopropyl-acrylamide)/poly(γ-glutamic acid)	[213]
	SOD3-overexpressing MSCs	[214,215]
	RM191A	[216]
renal diseases	hEC-SOD tempol	[217,218] [219,220,221], [222]
metabolic diseases	SODB	[223,224]
	nano-SOD	[225,226,227]
	MnTE-2-PyP^5+^	[228]
ocular diseases	SOD1	[229,230]
	rMnSOD	[231] *

rMnSOD = recombinant SOD2, PC-SOD = lecithinized Cu, Zn-SOD, O-HTCC-= O-(2-hydroxyl)propyl-3-trimethyl ammonium chitosan chloride, hEC-SOD = human recombinant SOD3, SODB = Cucumis melo L.C. derived SOD, nano-SOD = SOD1 encapsulated in poly-L-lysine (PLL50)-polyethylene glycol (PEG), cross-linked with a reducible cross-linker, TAT = human immunodeficiency virus type 1 (HIV) transactivator of transcription, SOD2m = SOD2 mimetic, MSCs = mesenchymal stromal cells,* clinical study.

**Table 3:** SOD-based compounds tested for potential therapeutic applications between 2012 and 2020.

SOD/SOD Donor	SOD Mimetics	Gene Therapy
CAR-modified liposomes fasudil plus SOD	[Fe(HPClNOL)Cl_2_]NO_3_	SOD3-overexpressing MSCs
gliadin SOD	MnTDE-2-ImP^5+^	
hEC-SOD	Calmangafodipir *	
MS-AOE^®^	EUK-134	
nano-SOD	EUK-207	
O-HTCC-SOD	GC4419 *	
PC-SOD	Nano-MnTnBuOE-2-PyP^5+^	
rMnSOD *	Mangafodipir *	
SOD-loaded thermo-sensitive hydrogel-poly(*N*-isopropyl-acrylamide)/poly(γ-glutamic acid)	mito-tempo	
SOD-loaded porous polymersome	Mn1	
SOD *	MnTE-2-PyP^5+^ *	
SOD1	MnTM-4-PyP^5+^	
SOD2	MnTnBuOE-2-PyP^5+^ *	
SOD2 by *Bacillus amyloliquefaciens* strain	MnTnHex-2-PyP^5+^	
SOD3	RM191A *	
SODB *	SOD2m	
TAT-SOD *	Tempol *	

rMnSOD = recombinant SOD2. PC-SOD = lecithinized Cu, Zn-SOD. O-HTCC- = O-(2-hydroxyl)propyl-3-trimethyl ammonium chitosan chloride. hEC-SOD = human recombinant SOD3. MS-AOE^®^ = Multi-modified stable anti-oxidant enzymes^®^. SODB = Cucumis melo L.C. derived SOD. nano-SOD = SOD1 encapsulated in poly-L-lysine (PLL50)-polyethylene glycol (PEG), cross-linked with a reducible cross-linker. TAT = human immunodeficiency virus type 1 (HIV) transactivator of transcription. SOD2m = SOD2 mimetic. MSCs = mesenchymal stromal cells. * also tested in clinical studies (https://clinicaltrials.gov or https://www.anzctr.org.au, last accessed on 19 March 2021).

**Table 4:** Examples of major exogenous natural SOD sources.

Terrestrial Plants	Microbial	Cyanobacteria	Chromista	Marine Plants
*Allium cepa* L.	*Anabaena Geobacillus* sp.	*Anabaena cylindrica*	*Lingulodinium polyedrum*	*Avicennia marina*
*Anacardium occidentale* L.	*Bacillus amyloliquefaciens*	*Anabaena variabilis Kutz*	*Minutocellus polymorphus*	*Bruguiera gymnorrhiza*
*Camellia sinensis*	*Bacillus subtilis*	*Cyanobacterium synechococcus*	*Nitzschia closterium*	*Enteromorpha linza*
*Cucumis melo L.C.*	*Brucella abortus*	*Microcystis aeruginosa*	*Thallassiosira weissflogii*	*Platymonas subcordiformis*
*Cucurbitamoschata* L.	*Caulobacter crescentus*	*Nostoc commune*		*Porphyridium cruentum*
*Fagopyrum tataricum*	*Escherichia coli*	*Nostoc PCC 7120*		*Sonneratia alba*
*Gossypium herbaceum* L.	*Haemophilus influenzae*	*Plectonema boryanum*		*Tetraselmis gracilis*
*Hordeum vulgare*	*Haemophilus parainfluenzae*	*Plectonema boryanum*		
*Luffa cylindrical*	*Lactobacillus fermentum*			
*Momordica charantia*	*Nodularia Aphanizomenon*			
*Momordicacharantia* L.	*Photobacterium leiognathi*			
*Nicotiana tabacum*	*Photobacterium phosphoreum*			
*Olea europaea* L.	*Photobacterium sepia*			
*Pisum sativum*	*Pseudomonas aeruginosa*			
Rosmarinus officinalis *				
*Saccharum* spp.				
Salvia officinalis *				
*Syzygium cumini*				
Thymus officinalis *				
*Vitis vinifera* L.				
*Zea mays* L.				

* Culinary herbs with SOD mimetic activity.

**Table 5:** A proposed SOD mimetic classification.

Cyclic Polyamines	MnPLED	MnPs	Salen-Mn Complexes	Metal-Based Compounds	Nitroxide
GC4419	calmangafodipir	MnTDE-2-ImP^5+^	EUK-134	[Fe(HPClNOL)Cl_2_]NO_3_	mito-tempo
Mn1	mangafodipir	MnTE-2-PyP^5+^	EUK-207	RM191A	tempol
		MnTM-4-PyP^5+^			
		MnTnBuOE-2-PyP^5+^			
		MnTnHex-2-PyP^5+^			

MnP = Mn porphyrin. MnPLED = Manganese pyridoxyl ethyldiamine derivatives.

## Data Availability

Not applicable.
